# Conserved Epithelial Remodeling Programs Underlie Pediatric Juvenile Colorectal Polyps Associated with Allergic Sensitization

**DOI:** 10.3390/ijms27177946

**Published:** 2026-09-07

**Authors:** María Belén Polo, Manuela Ilid, Viviana Bernedo, Paula Borobia, Lorena Menendez, Anabella Zosi, Cecilia Zubirí, Maximiliano Fernández Rivas, María Florencia Recalde, Barbara Virginia Aguilar Becher, Marcela García, Eugenia Altamirano, Luciana Guzmán, Martin Abba, Cecilia Muglia, Guillermo Docena

**Affiliations:** 1 Instituto de Estudios Inmunológicos y Fisiopatológicos-IIFP, Facultad de Ciencias Exactas, Universidad Nacional de La Plata, Consejo Nacional de Investigaciones Científicas y Técnicas (CONICET), La Plata 1900, Argentina; belenpolom@gmail.com (M.B.P.); manoletteilid@gmail.com (M.I.); guidoc@biol.unlp.edu.ar (G.D.); 2 Servicio de Gastroenterología, Hospital Interzonal de Agudos Especializado en Pediatría Sor María Ludovica, La Plata 1900, Argentinalorena.menendez@hotmail.com (L.M.); flor.recalde@hotmail.com (M.F.R.); lucianaguzman155@gmail.com (L.G.); 3 Sala de Alergia, Hospital Interzonal de Agudos Especializado en Pediatría Sor María Ludovica, La Plata 1900, Argentina; 4 Servicio de Patología, Hospital Interzonal de Agudos Especializado en Pediatría Sor María Ludovica, La Plata 1900, Argentina; 5 Centro de Investigaciones Inmunológicas Básicas y Aplicadas-CINIBA, Facultad de Ciencias Médicas de la Universidad Nacional de La Plata, La Plata 1900, Argentina; mcabba@gmail.com

**Keywords:** juvenile polyps, type 2 inflammation, IgE, epithelial transcriptomics, RNA-seq, colorectal cancer, pediatric gastroenterology, food allergy, *SERPINE1*, *SERPINB4*, *CHI3L1*

## Abstract

Juvenile colorectal polyps (JP) are the most common polypoid lesions of the colon in children and the leading cause of lower gastrointestinal bleeding in pediatric patients. Although histologically classified as benign hamartomatous lesions, they arise in a context of allergic sensitization and IgE-mediated inflammation, suggesting that the epithelial compartment may play an active role in their pathogenesis. To date, most studies have focused on the immune cell infiltrate, leaving the molecular programs operating within the epithelium largely unexplored. Whole-transcriptome RNA sequencing (RNA-seq) was performed on epithelial cells isolated from pediatric JP (*n* = 8) and paired tissue circumjacent to polyp (TCP) (*n* = 7) obtained from children with a history of rectal bleeding and IgE sensitization to food allergens. Differential expression, functional enrichment (GO, KEGG, GSEA), and cross-dataset comparison with TCGA colorectal adenocarcinoma (CRC) was conducted. Candidate genes were validated by RT-qPCR in independent samples, including colorectal cancer (CRC) and inflammatory bowel disease (IBD) tissue. Differential expression analysis identified 3273 differentially expressed genes (2342 upregulated, 931 downregulated in JP vs. TCP), with 220 genes exceeding 32-fold change, including *SERPINB3* (log2FC = 12.16), *MMP1* (log2FC = 10.19), *NMUR2* (log2FC = 10.08) and *CHI3L1* (log2FC = 8.87). JP epithelium displayed a coherent type 2 inflammatory signature, encompassing upregulation of the alarmin IL33, eosinophil-attracting chemokines (*CCL11*, *CCL24*), IgE receptor subunits (*FCER1A*, *FCER1G*), and a coordinately activated leukotriene and prostaglandin biosynthetic program (*ALOX5*, *ALOX5AP*, *PTGS2*). Concurrent alterations in epithelial identity were observed, including downregulation of absorptive enterocyte and intestinal stem cell markers (*CDX2*, *LGR5*, *ASCL2*) alongside upregulation of secretory and regenerative programs, including the ectopic gastric-type mucin *MUC5AC*. Tight junction dysregulation—notably upregulation of the pore-forming claudin *CLDN2* and loss of barrier-sealing claudins (*CLDN3*, *CLDN4*, *CLDN23*)—was consistent with impaired epithelial permeability. Pathway analyses confirmed activation of type 2 immune and extracellular matrix remodeling programs, with concurrent suppression of mitochondrial oxidative phosphorylation. Cross-dataset comparison with TCGA CRC data identified 381 co-upregulated and 87 co-downregulated genes shared between JP and CRC, including *SERPINE1*, *CXCL8*, *ICAM1*, and *MMP3*, several of which were associated with poorer disease-specific survival in CRC patients. RT-qPCR validation confirmed elevation of *CHI3L1* and *SERPINE1* in both JP and CRC, while *SERPINB4* appeared JP-specific. Epithelial cells from pediatric juvenile colorectal polyps associated with allergic sensitization display a comprehensive type 2 inflammatory transcriptome alongside profound alterations in lineage identity, barrier integrity, and metabolic programming. Partial convergence with CRC-associated gene expression programs—in the absence of histological dysplasia—suggests that chronic allergic inflammation activates conserved epithelial remodeling pathways shared across mucosal tissues. *CHI3L1*, *SERPINE1*, and *SERPINB4* are candidate biomarkers warranting validation in larger cohorts.

## 1. Introduction

Juvenile colorectal polyps (JP) represent the most frequent polypoid lesion of the colon in children, accounting for the majority of lower gastrointestinal bleeding in pediatric patients. These lesions are characterized histologically by dilated and inflamed glandular structures embedded in an edematous stroma rich in inflammatory cells, and are observed in patients with elevated total and allergen-specific IgE. Although JP are generally classified as benign and non-adenomatous, they are routinely removed endoscopically, largely due to their association with recurrent bleeding and concerns about long-term mucosal behavior.

The intestinal epithelium is not a passive barrier but an active immunological interface. Intestinal epithelial cells (IEC) sense microbial and dietary antigens, secrete alarmins, regulate immune cell recruitment, and modulate both innate and adaptive responses. In the context of allergic disease, IEC-derived signals—particularly the alarmin interleukin-33 (IL-33)—have been established as critical initiators of type 2 immune responses, acting on innate lymphoid cells (ILC2), mast cells, and eosinophils [1]. The epithelial layer therefore occupies a central position in the pathophysiology of food allergy and atopic gastrointestinal inflammation. Although allergic diseases affect anatomically distinct organs, accumulating evidence suggests that epithelial cells from different barrier tissues activate remarkably conserved transcriptional programs in response to chronic type 2 inflammation. Studies in airway, skin, and gastrointestinal diseases have demonstrated common epithelial responses characterized by alarmin production, disruption of barrier integrity, altered cellular differentiation, extracellular matrix remodeling, and metabolic adaptation. These observations raise the possibility that chronic allergic inflammation induces a core epithelial remodeling program that is largely independent of tissue location and reflects a shared mechanism of mucosal adaptation to persistent immune activation. Understanding whether juvenile colorectal polyps exhibit these conserved epithelial signatures may provide new insight into the molecular mechanisms linking allergic inflammation, epithelial plasticity, and tissue remodeling across barrier organs.

Type 2 inflammation in the gastrointestinal tract is well-characterized in conditions such as eosinophilic esophagitis and food protein-induced enterocolitis, and we recently reported that children with rectal bleeding and IgE sensitization showed solitary and pedunculated inflammatory hamartomatous colorectal polyps. However, most studies have focused on the immune cell compartment, leaving the contribution of the epithelium largely unexplored. Key type 2 mediators—including Th2 cytokines, IgE, eosinophil-attracting chemokines, and lipid mediators derived from the leukotriene and prostaglandin pathways—have been implicated in mucosal remodeling, epithelial permeability, and the modulation of intestinal stem cell niches. Whether JP-associated epithelial cells actively participate in the production and amplification of these signals remains an open question.

There is clinical interest concerning the molecular relationship between chronic intestinal inflammation and neoplastic transformation. Inflammation-associated colorectal carcinogenesis is well-established in the context of inflammatory bowel disease, and there is growing evidence that sustained inflammatory signaling can activate transcriptional programs that overlap with those observed in colorectal cancer (CRC) [2,3,4]. Juvenile polyps, arising in an inflammatory context in pediatric patients, represent an opportunity to investigate whether early epithelial reprogramming shares molecular features with CRC, even in the absence of histological dysplasia.

Recent single-cell transcriptomic analysis of pediatric colonic polyps by Deng et al. revealed distinct epithelial and stromal remodeling programs across solitary juvenile polyps, juvenile polyposis syndrome, and Peutz–Jeghers syndrome, highlighting alterations in epithelial differentiation, metabolic pathways, and tissue remodeling [5]. These findings provide an important transcriptomic framework for investigating epithelial reprogramming in juvenile polyps.

Building on our previous studies demonstrating local IgE production, food antigen-specific Th2 responses, epithelial production of CCL26, and the presence of a distinct tissue-associated microbiota enriched in bacterial taxa associated with intestinal inflammation and colorectal neoplasia [6], we sought to determine whether the epithelial compartment exhibits a corresponding transcriptional remodeling program. To address this question, we performed whole-transcriptome RNA sequencing of epithelial cells isolated from pediatric juvenile colorectal polyps and paired adjacent colonic tissue. We hypothesized that chronic allergic inflammation induces a conserved epithelial remodeling program characterized by alterations in barrier function, cellular differentiation, immune signaling, and metabolic pathways, potentially reflecting common adaptive mechanisms shared among allergic diseases affecting different mucosal barrier tissues. Furthermore, comparative analyses revealed significant overlap with transcriptional programs observed in colorectal cancer and eosinophilic nasal polyps, supporting the concept that chronic allergic inflammation activates conserved epithelial remodeling programs across distinct mucosal tissues while remaining biologically distinct from malignant transformation.

## 2. Results and Discussion

### 2.1. Global Transcriptomic Profiling Reveals Extensive Differential Expression Between JP and Adjacent Mucosa

**Figure 1:**
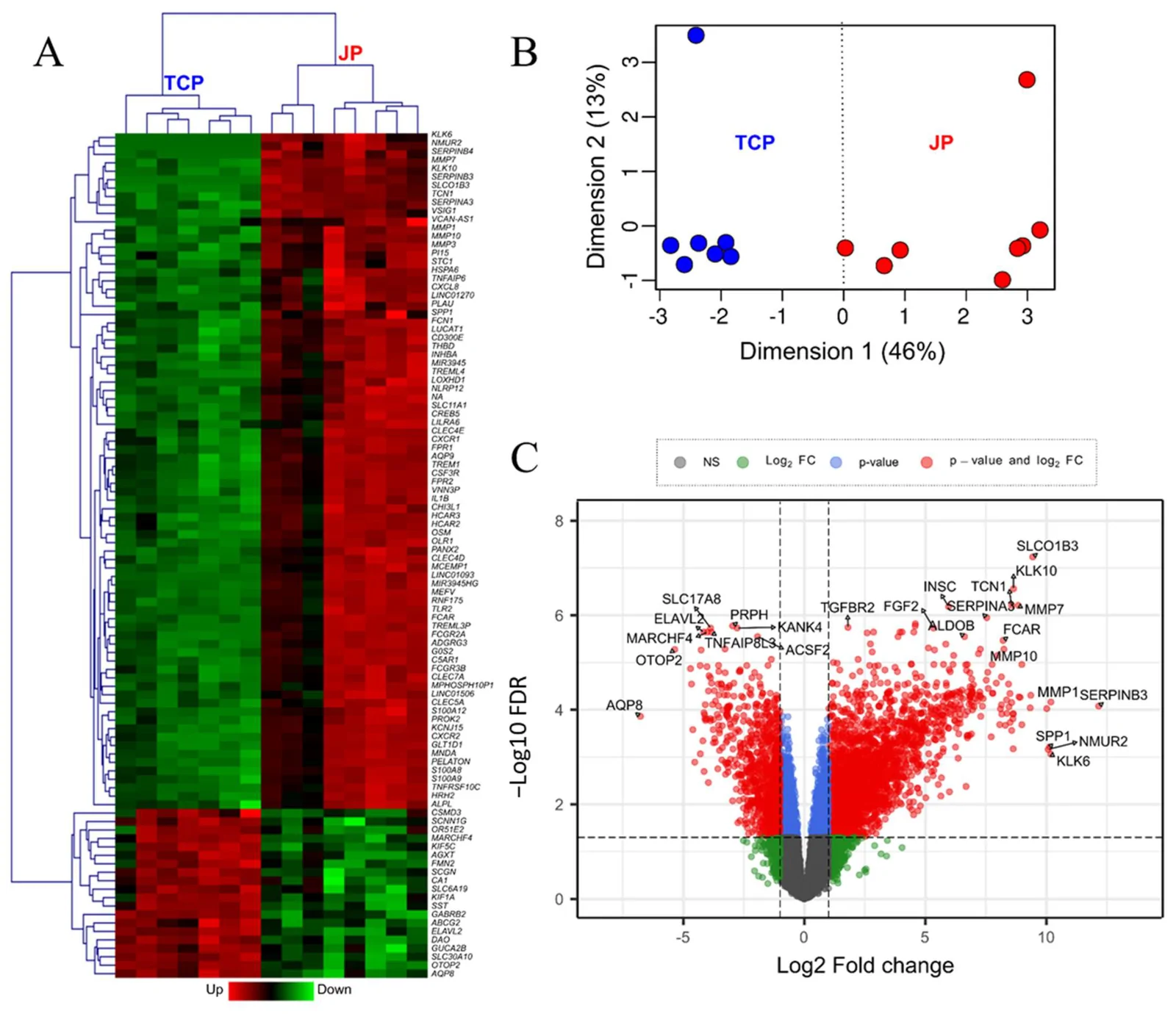
Global transcriptomic profiling of JP and TCP epithelial cells. (**A**) Heatmap of the top 100 differentially expressed genes (DEGs) identified by edgeR-QL. Columns represent individual samples; rows represent genes. Red: upregulated; green: downregulated. (**B**) MDS (Multidimensional Scaling) of all samples. Each point represents one sample; colors indicate tissue origin (red: juvenile polyp [JP]; blue: surrounding tissue [TCP]). MDS (46% variance) separates JP from TCP samples. (**C**) Volcano plot of all 17,589 quantified genes. Red: significant DEGs (log_2_FC > 1, FDR < 0.05); blue: significant but log_2_FC ≤ 1; grey: not significant. Key genes are labeled.

Differential expression analysis using edgeR-QL (FDR < 0.05, log2FC > 1) identified 3273 DEGs between JP and TCP, with 2342 genes upregulated and 931 downregulated in JP compared with tissue circumjacent to polyp (TCP) (Supplementary Data S1, Figure 1A,B). Unsupervised clustering based on these profiles showed a clear separation between JP and TCP samples, while hierarchical clustering of the top 100 DEGs confirmed a robust and consistent partitioning between conditions (Figure 1A,B). Notably, the magnitude of reprogramming was substantial; 220 genes displayed extreme differential expression with log2FC > 5 (>32-fold change), in some cases reaching values as high as 12 (~4096-fold change). This dramatic shift is further illustrated in the volcano plot (Figure 1C), which highlights highly significant transcripts such as *SERPINB3* (log2FC = 12.16), *MMP1* (log2FC = 10.19), *NMUR2* (log2 = 10.08) and *CHI3L1* (log2FC = 8.87), representing some of the most extensively regulated genes in the polyp epithelium.

### 2.2. Polyp Epithelium Exhibits Altered Lineage Identity and Epithelial Differentiation Programs

Analysis of lineage- and differentiation-associated genes revealed a pronounced shift in the cellular identity of polyp epithelium. Two key transcription factors governing intestinal epithelial differentiation, *CDX1* and *CDX2* (caudal-type homeobox proteins 1 and 2), were significantly downregulated in JP (log2FC = −1.10 and −1.16, respectively). As CDX2 is a central regulator of enterocyte identity and maintains the differentiated epithelial phenotype by activating genes such as *SI* (sucrase-isomaltase), its reduction is consistent with a loss of epithelial maturation.

In line with this, canonical absorptive enterocyte markers were significantly reduced in JP, including *VIL1* (log2FC = −1.33) and *ALPI* (log2FC = −2.69), as well as the enteroendocrine marker *CHGA* (log2FC = −3.88) and the tuft cell marker *DCLK1* (log2FC = −1.76). Conversely, secretory lineage genes were markedly upregulated, including *MUC2* (log2FC = 1.32), *LYZ* and *REG4* (log2FC = 3.37 and 4.59, respectively), consistent with an expansion of Paneth-like secretory cells. Strikingly, *MUC5AC* (log2FC = 6.03), a gastric-type mucin not normally expressed in the colon, was strongly upregulated, suggesting a metaplastic or stress-associated epithelial state.

Canonical intestinal stem cell markers were consistently downregulated in JP, including *LGR5* (log2FC = −2.10), *ASCL2* (log2FC = −1.96), *SMOC2* (log2FC = −3.50), and *PTK7* (log2FC = −1.13). Quiescent stem cell markers were also reduced (*LRIG1*, *MEX3A*, *TERT*). In contrast, *OLFM4* was upregulated (log2FC = 2.66), consistent with an immature or damage-associated regenerative epithelial state [7]. Key Notch and BMP signaling components were also altered: *HES1* (log2FC = −1.52), *BMP2* (log2FC = −1.55), and *ID2* (log2FC = −1.04) were downregulated, while *BMP4* (log2FC = 2.55) and the BMP antagonist *GREM1* (log2FC = 3.08) were upregulated, indicating disruption of canonical signaling axes that regulate lineage commitment (Table 1).

### 2.3. Barrier-Associated Gene Expression Is Disrupted in Polyp Epithelium

Analysis of tight junction genes revealed differential regulation of claudin family members in JP. *CLDN2* (log2FC = 5.70) and *CLDN1* (log2FC = 2.22) were markedly upregulated, while barrier-sealing claudins *CLDN3*, *CLDN4*, and *CLDN23* were significantly downregulated (log2FC = −1.14, −1.26, and −2.57, respectively). Since CLDN2 is a pore-forming claudin that increases paracellular permeability, its strong upregulation—combined with the loss of sealing claudins—is consistent with impaired barrier integrity in the polyp epithelium. These changes may facilitate antigen permeation and contribute to the inflammatory microenvironment observed in JP (Table 1).

### 2.4. Polyp Epithelium Displays a Robust Type 2 Inflammatory Transcriptional Signature

Among the most biologically informative findings was the identification of a strong and coherent type 2 inflammatory gene expression signature in JP epithelium. The transcript coding for alarmin IL-33, a cytokine secreted by IEC that initiates type 2 immune responses by activating ILC2, mast cells, and eosinophils, was significantly upregulated (log2FC = 2.57; approximately 6-fold), suggesting active epithelial participation in the orchestration of allergic inflammation.

Chemokines responsible for eosinophil recruitment were markedly elevated: *CCL11* (eotaxin-1; log2FC = 3.52) and *CCL24* (eotaxin-2; l log2FC = 2.10) were among the highly upregulated chemokines. *CCL22* (log2FC = 2.51), a CCR4 ligand that attracts Th2 lymphocytes and regulatory T cells, was also significantly increased, as was *CCL3* (MIP-1α; log2FC = 6.38), previously implicated in allergic asthma and atopic dermatitis. These findings strongly suggest that JP IEC actively recruits type 2 immune effectors to the polyp microenvironment.

IgE receptor subunit genes were upregulated in JP samples: both *FCER1G* (FcεRIγ; log2FC = 5.59) and *FCER1A* (FcεRIα; log2FC = 2.42) were differentially expressed. Although these subunits are not typically expressed by IEC, the enrichment protocol used yields a cell preparation enriched but not strictly purified for epithelial cells, and the presence of resident immune cells in the isolated fraction cannot be excluded. Nevertheless, the expression of these genes underscores the IgE-mediated inflammatory character of the JP microenvironment.

The leukotriene biosynthesis pathway was coordinately upregulated in JP, including *ALOX5* (5-lipoxygenase; log2FC = 3.85), *ALOX5AP* (5-lipoxygenase-activating protein; log2FC = 4.51), *CYSLTR2* (cysteinyl leukotriene receptor 2; log2FC = 3.06), and *DPEP2* (dipeptidase 2; log2FC = 4.89). The cyclooxygenase-2 gene *PTGS2* (COX-2; log2FC = 5.74) was also significantly elevated, indicating concurrent activation of prostaglandin biosynthesis. Toll-like receptor 4 (*TLR4*; log2FC = 5.14), a pattern-recognition receptor involved in innate immune activation and previously linked to allergen sensitization to dust mites and nickel [8,9], was markedly upregulated. Additionally, the genes most highly overexpressed in JP included *SERPINB3* (log2FC = 12.16), *CHI3L1* (log2FC = 8.87), and *SERPINB4* (log2FC = 8.16), which are established markers of type 2 inflammation, tissue remodeling, and allergic disease [10,11]. A summary of the most relevant type 2 inflammatory DEGs is provided in Table 2.

### 2.5. Gene Set Enrichment Analysis Confirms Activation of Type 2 Immune Pathways and Metabolic Reprogramming

**Figure 2:**
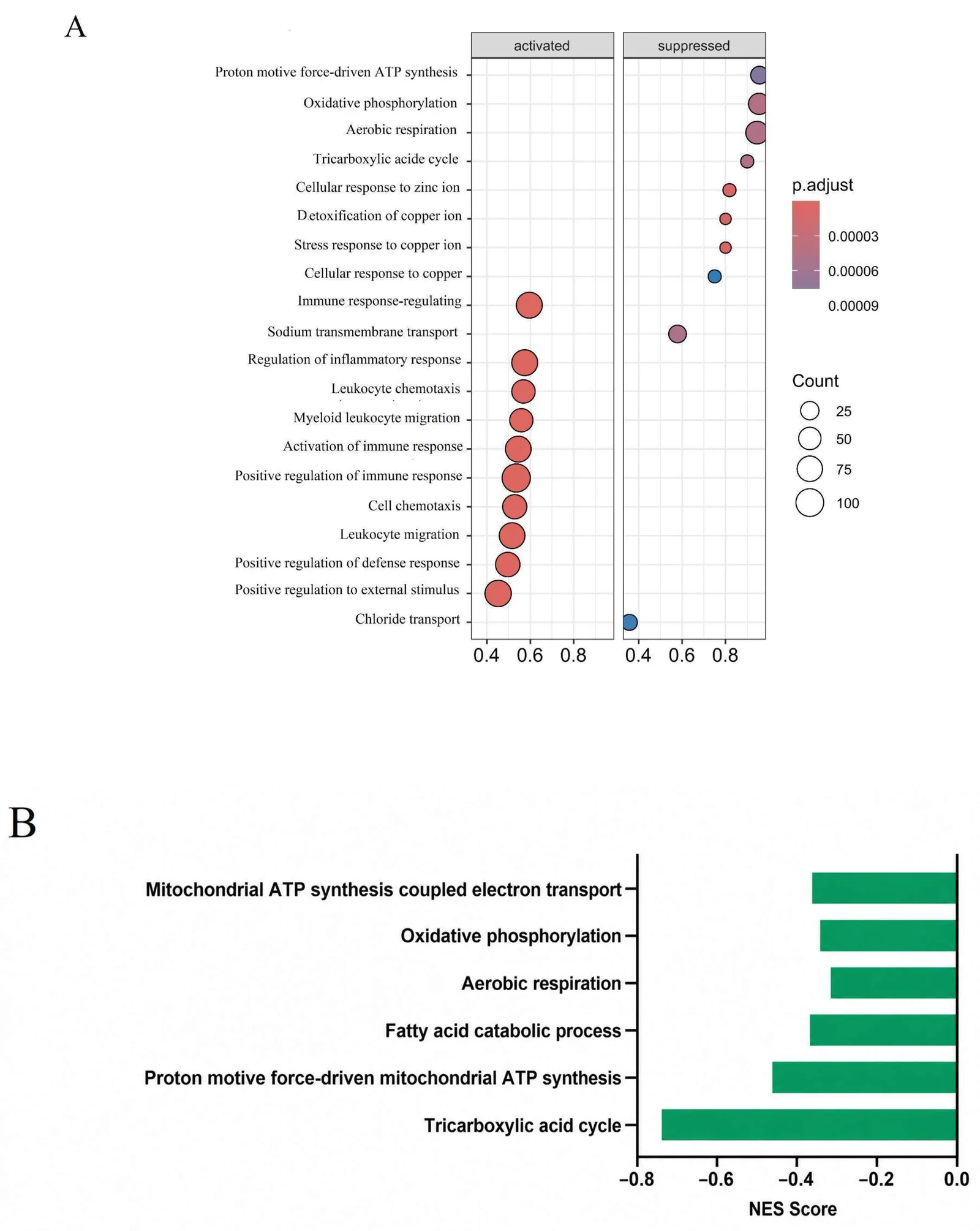

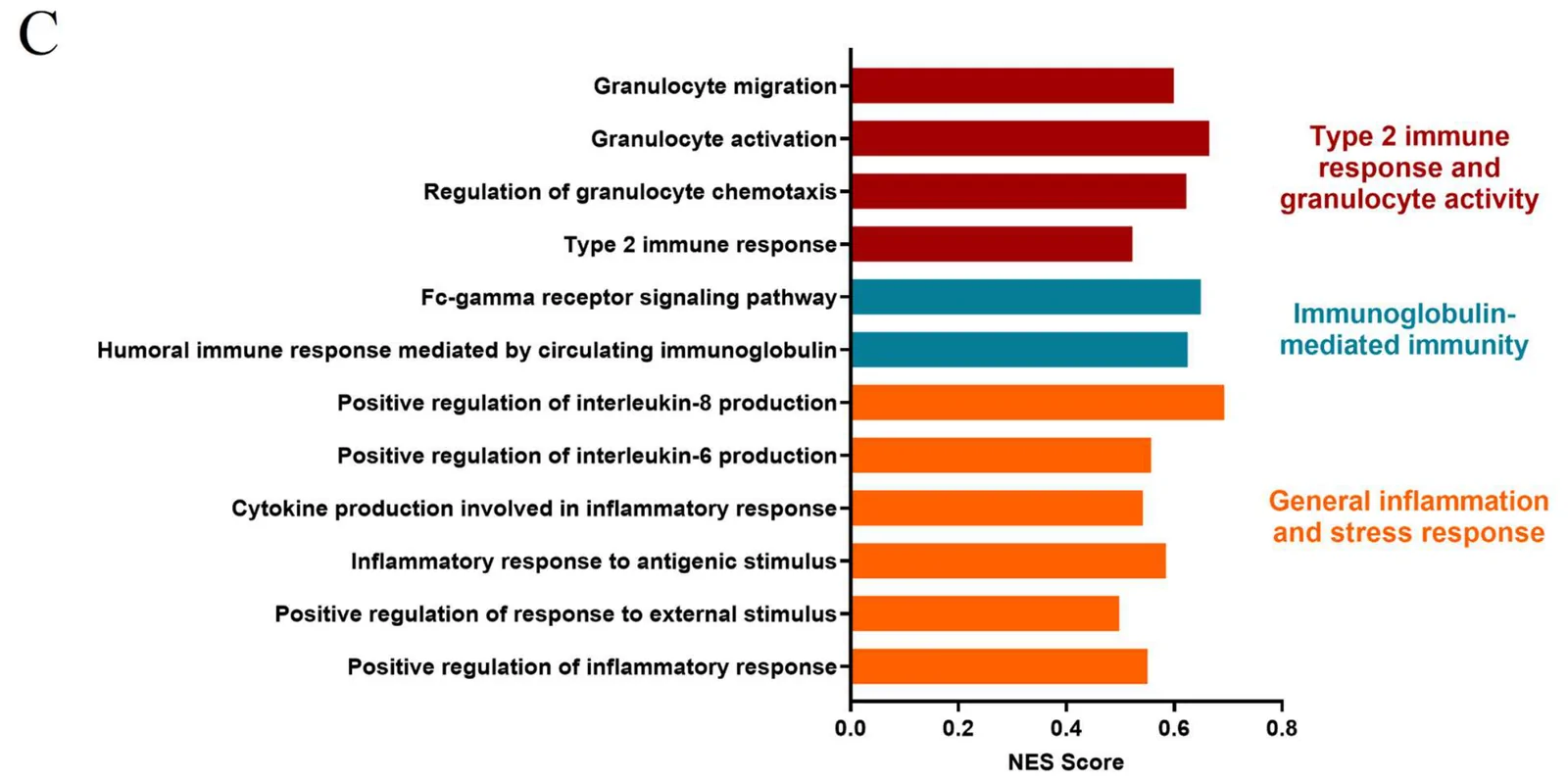
Gene Set Enrichment Analysis in JP versus TCP. (**A**) Dot plot summarizing the top activated and repressed GO terms. The x-axis indicates the GeneRatio, dot size represents the number of genes (count), and dot color corresponds to the adjusted *p*-value. (**B**) Bar chart of selected repressed GO terms (NES < 0, adjusted *p* < 0.05), predominantly related to mitochondrial oxidative metabolism. (**C**) Bar chart showing selected activated GO terms (GSEA, NES > 0, adjusted *p* < 0.05), into general inflammation/stress (red), immunoglobulin-mediated immunity (blue), and type 2 immunity/granulocyte activity (orange).

GSEA identified 709 activated (NES > 0) and 63 repressed (NES < 0) GO pathways (adjusted *p* < 0.05), together with 24 activated and 6 repressed KEGG pathways in JP versus TCP (Figure 2). The complete list of the GSEA results are provided in Supplementary Data S1.

Remarkably, the majority of repressed pathways in JP were associated with mitochondrial oxidative metabolism: oxidative phosphorylation, the tricarboxylic acid cycle, and ATP synthesis were among the most consistently downregulated GO and KEGG terms. This pattern is consistent with a metabolic shift away from oxidative phosphorylation, potentially toward aerobic glycolysis, which has been described in cells under conditions of hypoxia, chronic inflammation, or oncogenic transformation—all of which may be relevant to the JP microenvironment [12] (Figure 2A,B).

Remarkably, several pathways showed overexpressed genes related with an inflammation and stress response, including type-2 immunity and granulocyte chemoattraction (Figure 2C).

Consistent with the GSEA results, visualization of individual differentially expressed genes revealed two coordinated transcriptional programs that clearly distinguished JP from TCP (Supplementary Figure S1). The epithelial remodeling signature was characterized by increased expression of secretory, regenerative, and barrier-remodeling genes, together with reduced expression of genes associated with absorptive differentiation, intestinal stem cell identity, and barrier integrity. In parallel, JP displayed a highly consistent type 2 inflammation-associated signature, encompassing alarmins, eosinophil- and Th2-associated chemokines, IgE receptor components, and genes involved in leukotriene and prostaglandin pathways. These findings further support the coexistence of epithelial reprogramming and type 2 inflammation as major features of the JP transcriptome.

### 2.6. Polyp Epithelial Transcriptome Partially Converges with Colorectal Cancer-Associated Gene Expression Signatures and Biological Programs

**Figure 3:**
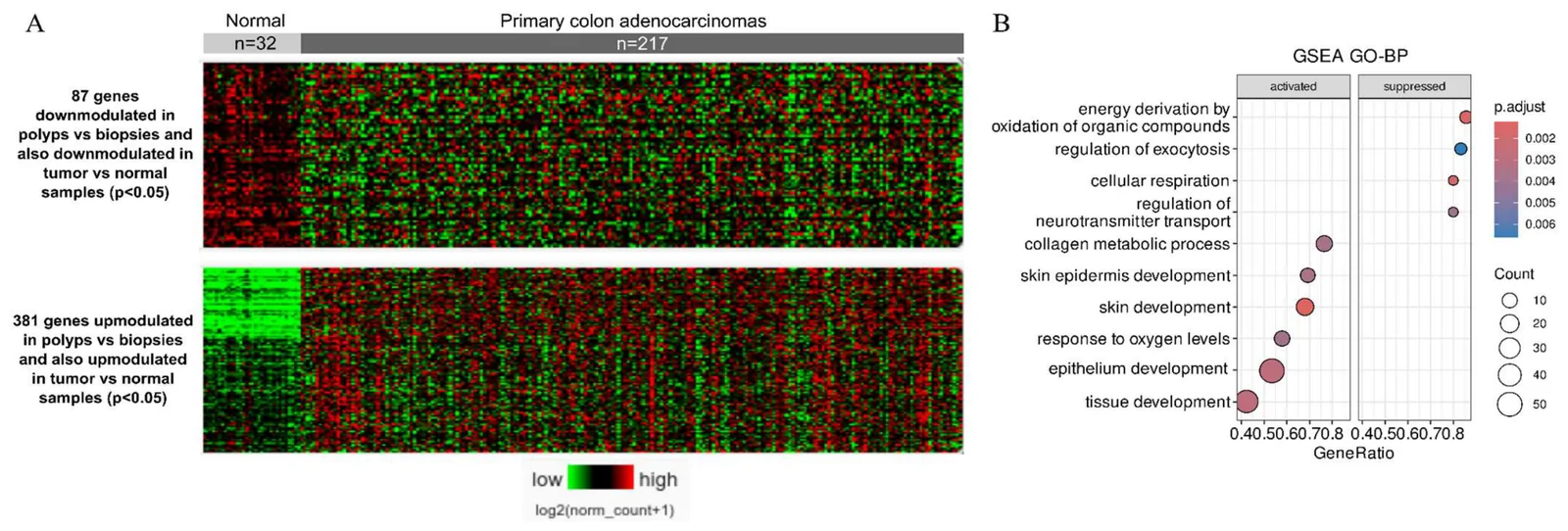

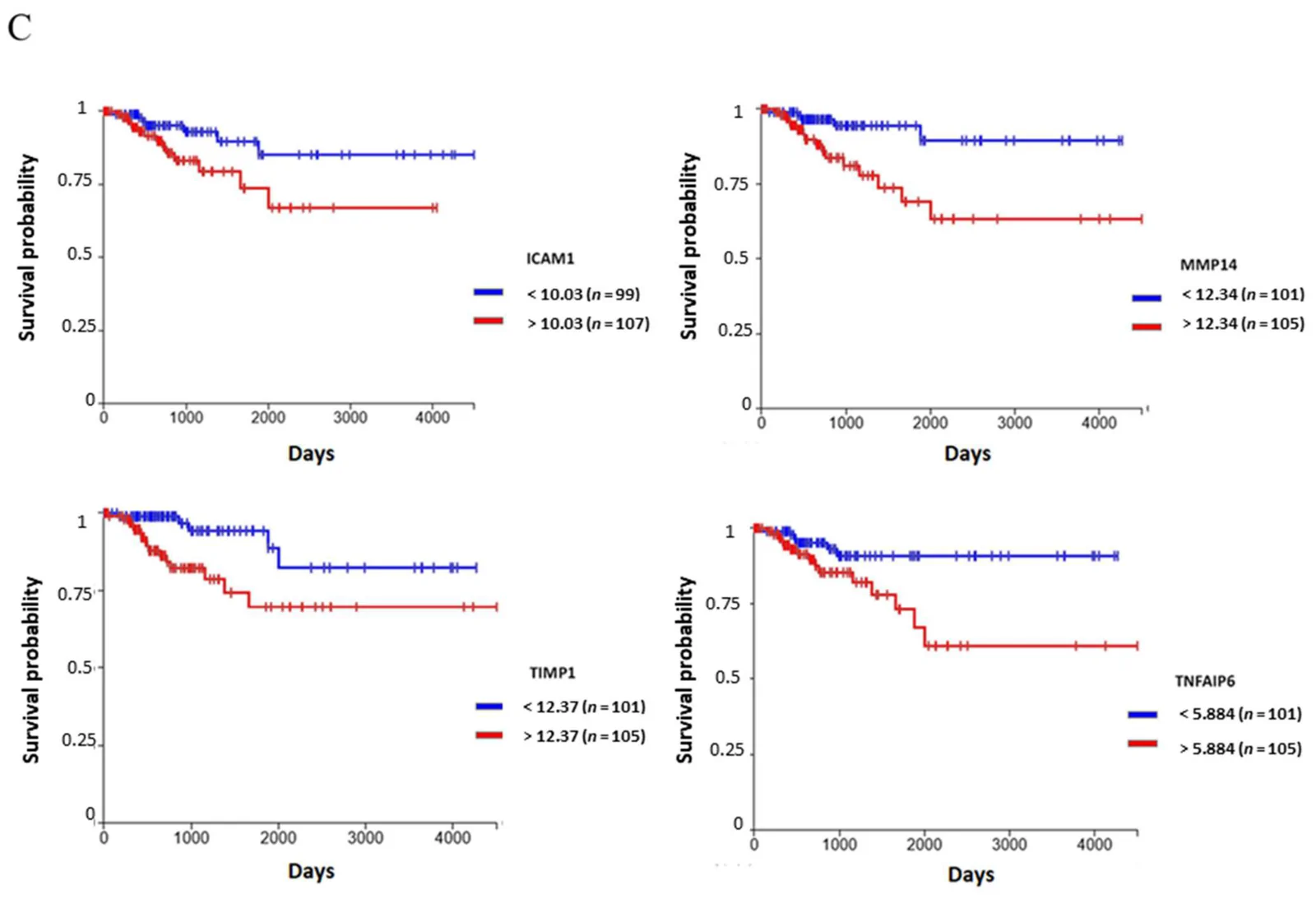
Cross-comparison of JP DEGs with TCGA colorectal adenocarcinoma data. (**A**) Heatmap of genes co-downregulated (top panel; *n* = 87) and co-upregulated (bottom panel; *n* = 381) in both JP versus TCP and CRC versus normal colonic tissue. Columns: TCGA samples (normal, *n* = 32; tumor, *n* = 217); rows: genes; color scale: log_2_(norm_count + 1). (**B**) Dot plot summarizing the top activated and repressed GO terms among the co-regulated genes. (**C**) Kaplan–Meier disease-specific survival curves for CRC patients stratified by high versus low expression of *ICAM1*, *MMP14*, *TIMP1*, and *TNFAIP6* (median as cutoff). Higher expression is associated with poorer survival in all four cases.

Cross-comparison of the JP transcriptomic profile with TCGA colorectal adenocarcinoma data identified 468 genes showing concordant regulation in both conditions, including 381 co-upregulated and 87 co-downregulated genes (Figure 3A). To further characterize the biological significance of these 468 coordinately regulated genes, GSEA was performed using GO Biological Process annotations (Figure 3B). Co-upregulated genes were enriched in functionally related processes involving epithelial and tissue remodeling, including epithelium/epidermis development, collagen metabolism, tissue morphogenesis, and responses to oxygen levels. Although several of these overlapping GO categories are annotated as “skin” or “epidermis development”, they largely comprise genes involved in epithelial differentiation, cell adhesion, extracellular matrix organization, and tissue repair rather than reflecting a skin-specific transcriptional program. Conversely, co-downregulated genes were predominantly associated with cellular respiration and energy derivation through oxidation of organic compounds, indicating a shared suppression of oxidative metabolic programs. Together, these findings suggest that JP and CRC partially converge not only at the level of individual genes but also in coordinated epithelial remodeling and metabolic reprogramming (Figure 3B).

Among the most notable shared upregulated genes, *SERPINE1* (PAI-1; log2FC = 4.42 in JP; *p* = 1.316 × 10^−11^ in TCGA) encodes plasminogen activator inhibitor-1, a key regulator of fibrinolysis and tissue remodeling with well-documented pro-tumorigenic functions in CRC, including promotion of angiogenesis and activation of P38-MAPK signaling [13,14]. Its expression in JP correlated with co-upregulation of *ITGA5* (log2FC = 5.36), *MMP19* (log2FC = 4.72), and *ADAMTS4* (log2FC = 3.25), genes previously shown to be co-expressed with *SERPINE1* in CRC datasets and collectively associated with ECM remodeling, cell adhesion regulation, and angiogenesis (Figure 3A) [15].

Additional shared upregulated genes included matrix metalloproteinases *MMP1* (log2FC = 10.19) and *MMP3* (log2FC = 10.02), which facilitate matrix degradation and tumor invasion; *CXCL8* (IL-8; log2FC = 8.10), a pro-inflammatory and pro-angiogenic chemokine associated with tumor immune exclusion; *FOXQ1* (log2FC = 5.96), a transcription factor linked to epithelial-to-mesenchymal transition and CRC metastasis; *TGFBI* (log2FC = 4.89), a TGF-β-induced protein regulating ECM adhesion; and *ICAM1* (log2FC = 4.67), an intercellular adhesion molecule implicated in both inflammatory and metastatic processes. Survival analysis using TCGA data and the UCSC Xena data base revealed that elevated expression of the convergent genes coding for *ICAM1, MMP14, TIMP1*, and *TNFAIP6* in the primary tumor was associated with significantly poorer disease-specific survival in CRC patients (Kaplan–Meier analysis, Figure 3C) (Table 3).

### 2.7. RT-qPCR Validation Confirms Overexpression of Candidate Genes in Independent JP Samples

**Figure 4:**
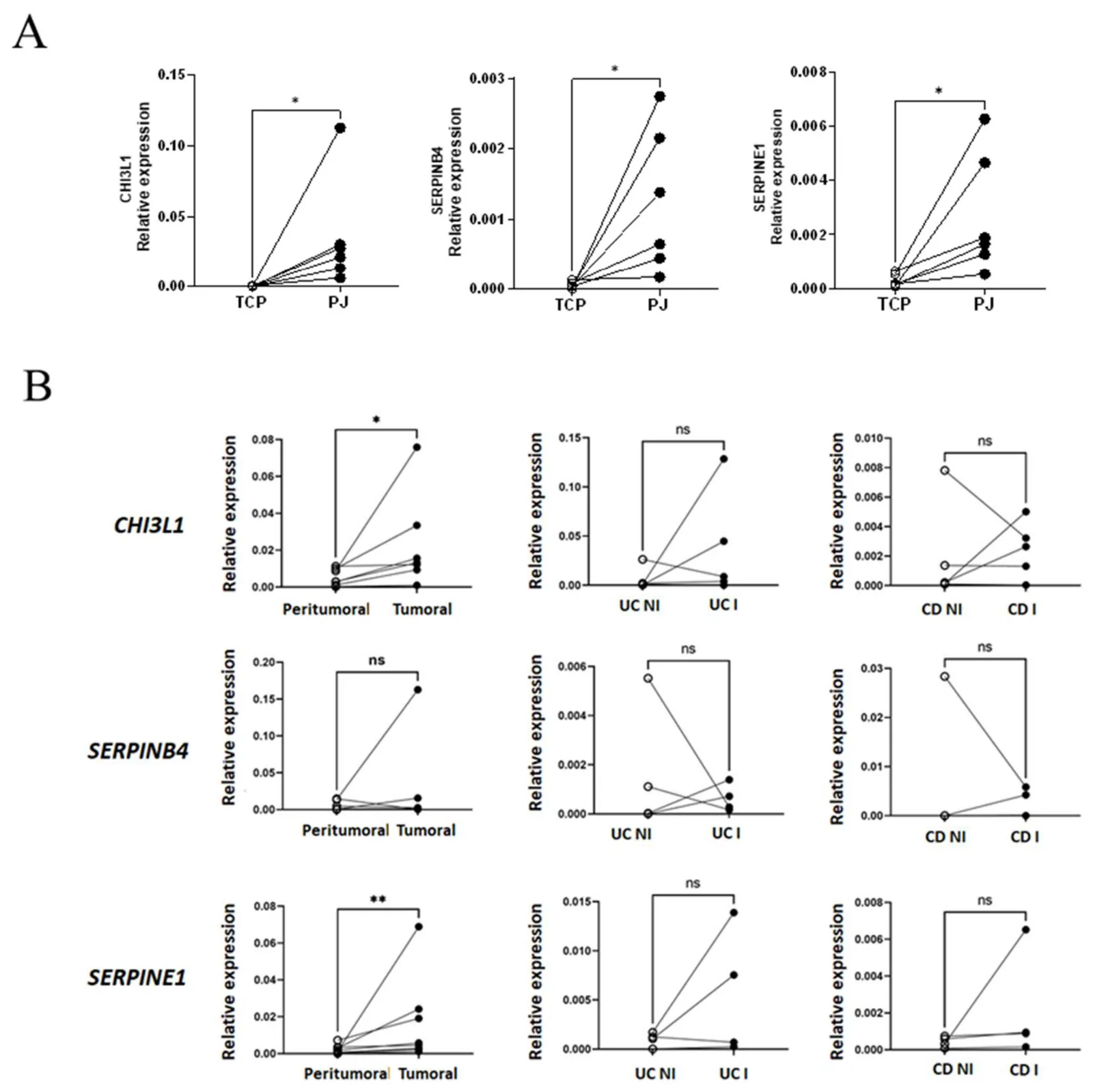
RT-qPCR validation of candidate genes. (**A**) Relative expression of *CHI3L1*, *SERPINB4*, and *SERPINE1* in independent JP (*n* = 6) and TCP (*n* = 6) IEC samples, normalized to *GAPDH*. Wilcoxon test; *p* = *0.03* for all three comparisons. (**B**) Relative expression of the same genes in CRC tumor versus peritumoral tissue (*n*= 7 pairs) and in inflamed versus non-inflamed mucosa from UC (*n* = 4) and CD (*n* = 4) patients. *CHI3L1* and *SERPINE1* are elevated in CRC (*p* = 0.0156 and *p* = 0.0078, respectively) but not in IBD. *SERPINB4* shows no significant difference in CRC or IBD. * *p* ˂ 0.05; ** *p* ˂ 0.01.

To validate the RNA-seq findings, expression of three selected candidate genes was assessed by RT-qPCR in six independent IEC samples from JP and paired TCP (distinct from those used for RNA-seq). The genes selected were: *CHI3L1* and *SERPINB4*, representing the type 2 inflammatory signature, and *SERPINE1*, representing the CRC-associated signature. All three genes showed significant overexpression in JP relative to TCP (Wilcoxon test: *p* = 0.03 for each), confirming the robustness of the transcriptomic findings in an independent sample set (Figure 4A).

To determine whether these expression changes were specific to the JP inflammatory context or reflected a broader inflammatory response, transcript levels were assessed in additional clinical cohorts: seven paired CRC tumor and peritumoral tissue samples, four UC samples (inflamed and non-inflamed zones), and four CD samples (inflamed and non-inflamed zones) (Figure 4B). In CRC, *CHI3L1* and *SERPINE1* were significantly elevated in tumor tissue compared with peritumoral samples (Wilcoxon test: *p* = 0.0156 and *p* = 0.0078, respectively), consistent with their established roles in CRC. In contrast, *SERPINB4* did not show significant differences between tumor and peritumoral tissue, suggesting that its upregulation may be specific to the JP epithelial context. Notably, none of the three genes exhibited significant differential expression in either UC or CD samples (Figure 4B), indicating that their overexpression is not a general feature of intestinal inflammation but rather appears to be characteristic of JP and, for two genes, of CRC.

Taken together, our findings position the epithelium as a central orchestrator of type 2 immunity in the context of pediatric solitary juvenile polyps. This concept is consistent with accumulating evidence from allergic diseases of barrier tissues, including atopic dermatitis and eosinophilic gastrointestinal disorders, where epithelial-derived alarmins such as IL-33, IL-25, and TSLP act as upstream regulators of type 2 immune polarization [16,17,18]. In this framework, the strong upregulation of *IL33* and eosinophil-attracting chemokines (*CCL11*, *CCL24*) observed in our dataset supports a model in which epithelial cells actively initiate and sustain local allergic inflammation, rather than merely responding to infiltrating immune cells. We previously reported that the inflammatory polyp tissue is dominated by eosinophils, with an increased production of the epithelial-derived eotaxin-3 [19,20].

The transcriptional activation of leukotriene and prostaglandin pathways further reinforces this interpretation. Previous studies have demonstrated that epithelial cells can contribute to lipid mediator production in inflammatory contexts, amplifying type 2 responses through paracrine signaling loops involving eosinophils, mast cells, and innate lymphoid cells [21,22,23]. The coordinated upregulation of *ALOX5*, *ALOX5AP*, *CYSLTR2*, and *PTGS2* in our cohort suggests that similar mechanisms may operate in juvenile polyps, potentially contributing to both inflammation and tissue remodeling.

Our data also highlight a profound alteration in epithelial differentiation and identity. The downregulation of canonical stem cell and absorptive markers, together with the upregulation of secretory and regenerative programs, is reminiscent of epithelial remodeling observed in chronic inflammatory conditions such as inflammatory bowel disease and allergic airway disease [24,25]. Notably, IL-13-driven epithelial reprogramming has been shown to promote goblet cell hyperplasia, mucus production, and barrier dysfunction [26,27], features that are partially recapitulated in our transcriptomic analysis. The aberrant expression of *MUC5AC*, a gastric-type mucin, further suggests the presence of a stress-induced or metaplastic epithelial state, which has been associated with chronic inflammation and early neoplastic transformation in gastrointestinal tissues [28].

The coordinated expression pattern observed across individual JP samples further supports the concept that epithelial remodeling is not restricted to isolated differentially expressed genes but represents a broader transcriptional program occurring alongside type 2 inflammation (Supplementary Figure S1).

Barrier dysfunction emerges as another key component of the JP epithelial phenotype. The imbalance between pore-forming and sealing claudins, particularly the marked upregulation of *CLDN2* and downregulation of *CLDN3*/*4*/*23*, is consistent with increased epithelial permeability. Similar alterations have been reported in food allergy and inflammatory bowel disease, where barrier disruption facilitates antigen translocation and perpetuates immune activation [29,30]. In this context, our findings support a feed-forward model in which epithelial barrier defects enhance exposure to dietary and microbial antigens, thereby reinforcing local type 2 inflammation.

One of the most intriguing aspects of our study is the partial convergence between the JP epithelial transcriptome and colorectal cancer-associated gene expression programs. Importantly, functional enrichment analysis of the 468 genes coordinately regulated in JP and CRC extended this convergence beyond individual genes, revealing shared enrichment of epithelial and tissue remodeling programs, including epithelial differentiation, extracellular matrix and collagen organization, and tissue morphogenesis, together with suppression of oxidative metabolic processes. The enrichment of GO categories annotated as skin or epidermis development likely reflects the extensive reuse of epithelial structural, differentiation, adhesion, and repair programs across barrier tissues rather than acquisition of a tissue-specific cutaneous phenotype. Chronic inflammation is a well-established driver of tumorigenesis [31], and several of the genes identified in our analysis—including *SERPINE1*, *MMPs*, *CXCL8*, and *ICAM1*—have been implicated in extracellular matrix remodeling, angiogenesis, and tumor progression [32,33,34]. The overexpression of *SERPINE1*, in particular, has been consistently associated with poor prognosis and pro-tumorigenic signaling in colorectal cancer [15,35]. Its presence in solitary non-adenomatous juvenile polyps suggests that elements of these pathways may be activated early, even in histologically benign lesions.

Importantly, this transcriptional overlap does not imply that juvenile polyps are premalignant lesions per se, but rather that they may share common inflammatory and remodeling programs with neoplastic processes.

The mechanisms responsible for the activation of these cancer-associated transcriptional programs in histologically benign juvenile polyps remain incompletely understood. One potential contributor is the interaction between epithelial cells and the tissue-associated microbiota. In a recent study from our group [6], juvenile polyps from food-sensitized children harbored a distinct microbiota enriched in bacterial taxa previously associated with intestinal inflammation, colorectal adenomas and colorectal cancer, including *Escherichia*, *Fusobacterium*, *Enterocloster* and *Akkermansia*. Although the present transcriptomic analysis cannot establish a causal relationship, these observations support the hypothesis that epithelial remodeling and microbial dysbiosis reinforce one another during chronic allergic inflammation. Type 2 cytokine-mediated impairment of epithelial differentiation and barrier integrity may facilitate the establishment of a dysbiotic mucosa-associated microbiota, whose microbial products could in turn sustain epithelial activation through pathways involved in innate immune signaling, wound repair, extracellular matrix remodeling, metabolic reprogramming and cell survival. Such bidirectional interactions may help explain why juvenile polyps exhibit transcriptional programs that overlap with those described in colorectal cancer while remaining histologically benign. Rather than reflecting early malignant transformation, these signatures may represent a chronic epithelial adaptation driven by the combined effects of allergic inflammation and persistent microbial stimulation. Future studies integrating epithelial transcriptomics, spatial microbiome profiling and functional analyses will be essential to determine how tissue-associated microbial communities contribute to the conserved epithelial remodeling program that characterizes allergic juvenile colorectal polyps and other chronic inflammatory diseases of barrier tissues.

Similar observations have been made in other chronic inflammatory conditions, where sustained epithelial stress and immune activation lead to the activation of pathways that, in different contexts, contribute to tumorigenesis [31,36]. Interestingly, comparative analysis of our dataset with publicly available RNA-seq data from eosinophilic nasal polyps from patients with chronic rhinosinusitis (GSE136825) revealed remarkable overlap in biological processes despite the distinct anatomical locations of the lesions [37]. The whole-transcriptome RNA sequencing of both polyps showed chronic type 2 inflammation, epithelial remodeling, impaired barrier function, and extracellular matrix reorganization. These similarities suggest that persistent allergic inflammation induces a conserved epithelial response across mucosal tissues. The literature does not reflect that these nasal inflammatory lesions can activate tumor-associated transcriptional programs that progress to tumors. Transcriptomic analyses have revealed that eosinophilic nasal polyps activate several biological pathways also observed in epithelial tumors—including extracellular matrix remodeling, angiogenesis, wound-healing responses, and NF-κB/STAT3 signaling—yet they lack the genomic instability, recurrent driver mutations, dysplasia, and uncontrolled proliferative programs that characterize malignant transformation [37]. The comparison of the whole-transcriptome RNA sequencing of eosinophilic nasal polyps in patients with chronic rhinosinusitis vs. healthy inferior turbinate nasal tissue from healthy patients revealed shared enriched biological pathways with the JP transcriptomic analysis that are upregulated or downregulated. Notably, both transcriptomes exhibited epithelial lineage reprogramming, suppression of oxidative phosphorylation, extracellular matrix remodeling, activation of inflammatory type 2 cytokine networks, barrier tissue dysfunction and induction of genes associated with epithelial repair. An additional factor that may contribute to the conserved epithelial remodelling observed across mucosal tissues is the altered tissue-associated microbiota, previously mentioned. Chronic rhinosinusitis with nasal polyps has been associated with *Staphylococcus aureus* infection, supporting the concept that epithelial activation occurs in close association with local dysbiosis [38]. Together with our previous observation that juvenile polyps harbour a distinct tissue-associated microbiota enriched in bacteria associated with intestinal inflammation and colorectal neoplasia, these findings suggest that dysbiosis may represent a common environmental driver reinforcing epithelial inflammatory remodelling across anatomically distinct barrier tissues.

Collectively, these observations suggest that hamartomatous juvenile polyps constitute an attractive human model for investigating epithelial plasticity induced by chronic allergic inflammation. Furthermore, comparison with eosinophilic nasal polyps indicates that conserved transcriptional programs operate across distinct mucosal tissues and may represent general mechanisms of barrier remodelling rather than early stages of malignant transformation.

Deng et al. described different non-adenomatous polyps and made a single-cell transcriptomic atlas from three pediatric colonic polyp subtypes—solitary juvenile polyps (SJP), juvenile polyposis syndrome (JPS), and Peutz–Jeghers syndrome (PJS). Solitary juvenile polyps analyzed in our cohort are most directly comparable to the SJP category in that study, which is similarly defined as inflammatory, structurally rearranged, and non-syndromic. Cross-referencing our 3273 DEGs with the gene-level signatures reported across all three subtypes revealed extensive and biologically coherent overlap. However, our RNA-seq was performed on whole epithelial biopsies rather than dissociated single cells, which may indicate a genuine biological difference between cohorts. Even so, there are 8 convergent pathways, including EMT-like colonocyte program, metabolic suppression with coincident genes downregulated and loss of epithelial identity with several absorptive colonocyte genes reduced [5].

Finally, our findings should be interpreted in the context of emerging concepts linking mucosal immunity, environmental exposure, and epithelial biology. The gastrointestinal tract is continuously exposed to dietary antigens and microbiota-derived signals, and epithelial cells integrate these inputs to shape immune responses [39,40]. In allergic individuals, this balance is altered, leading to exaggerated type 2 responses and impaired tolerance. The epithelial transcriptional program described here is consistent with such a dysregulated state and provides a molecular framework to understand how food sensitization may be linked to local tissue pathology.

The limitations of this study include the relatively small sample size, inherent to the limited availability of pediatric juvenile polyp specimens suitable for epithelial isolation and RNA sequencing, which may restrict the statistical power and generalizability of the findings. In addition, although the epithelial enrichment procedure yielded cell preparations enriched in epithelial cells, the purity of these fractions was not quantitatively assessed; therefore, a contribution from residual immune or stromal cells to some of the observed transcriptional signals cannot be excluded. Another limitation concerns the cross-dataset comparison with colorectal cancer, which involved adult colorectal cancer (CRC) samples from The Cancer Genome Atlas (TCGA), whereas the juvenile polyp (JP) samples were obtained from pediatric patients. Importantly, TCGA colorectal adenocarcinoma profiles were generated from bulk tumor tissue containing a mixture of epithelial, stromal, and immune cell populations, whereas our JP transcriptomic data were generated from epithelium-enriched pediatric samples. Consequently, part of the transcriptional overlap observed between the two datasets may reflect stromal or immune cell infiltration in the bulk TCGA tumors rather than a shared epithelial-intrinsic transcriptional program. Furthermore, age-related differences in epithelial biology and gene expression may contribute to some of the observed similarities or differences and should therefore be considered when interpreting this cross-dataset comparison. All patients with JP were recruited at a single clinical center, potentially introducing selection bias and limiting the broader applicability of the findings to other pediatric populations. Finally, although selected transcriptomic findings were independently validated by RT-qPCR, protein-level validation was not performed. Future studies incorporating larger multicenter pediatric cohorts, quantitative assessment of epithelial cell purity, and validation of key candidates at the protein and functional levels will be important to confirm and extend these observations.

## 3. Materials and Methods

### 3.1. Patient Cohort and Sample Collection

Fresh intestinal specimens were obtained from pediatric patients undergoing colonoscopy at the Children Hospital Sor María Ludovica of La Plata (Argentina). JP samples (*n* = 8) were collected from macroscopically identifiable polyps; paired surrounding colonic mucosa (TCP) (*n* = 7) was obtained from endoscopically normal tissue from the same patients. All patients had a clinical history of rectal bleeding and elevated serum total and specific IgE levels. No clinical symptoms of allergy were reported in these patients and all skin tests were negative (Skin Prick test). We detected IgE+ cells by flow cytometry in all polyps (Table 4). Written informed consent was obtained from patients or parents, and the study protocol was approved by the Institutional Protocol Review Committee of the Sor María Ludovica Children’s Hospital (#389-2023, #389-2025, approved in March 2023 and revalidated in July 2025). Patient data, including age (2–6 years old), sex (6 males and 4 females), allergy history, and endoscopic findings, were recorded.

### 3.2. Epithelial Cell Isolation

Intestinal epithelial cells were isolated from fresh biopsy specimens using a chelation-based protocol optimized for pediatric endoscopic material. Briefly, biopsies were washed in ice-cold phosphate-buffered saline and incubated in EDTA-containing buffer to dissociate the epithelial layer. The resulting cell suspension was enriched for epithelial cells and assessed for viability and purity prior to RNA extraction. Total RNA was extracted using the RNA Easy Fast Tissue/Cell Kit (TIANGEN Biotech, Beijing, China) and quality was assessed by Agilent 2100 Bioanalyzer (Agilent Technologies, Santa Clara, CA, USA); samples with RNA integrity number (RIN) ≥ 8 were included.

### 3.3. RNA Sequencing and Data Processing

RNA samples with RNA integrity number (RIN) over 8 were considered for RNA sequencing. The RNA samples were processed for directional RNA-seq library construction using the Illumina Stranded Total RNA with Ribo-Zero Plus Library Prep kit (BioSystems, Buenos Aires, Argentina) according to the manufacturer’s protocol. We performed 100 nt paired-end sequencing using an Illumina NextSeq 2000 platform at the National University of La Plata and obtained ~85 million reads per sample with 92% >Q30. The RNAseq raw data were submitted to the Sequence Read Archive (SRA) under BioProject accession number PRJNA1485045. The raw short-read sequences were quality-checked and trimmed to remove adapters and low-quality bases using the Rfastp 1.14.0 R/Bioconductor package. The preprocessed reads were then aligned and mapped to the human genome reference GRCh38 using the Subread aligner algorithm provided by the Rsubread 2.18.0 R/Bioconductor package. The aligned reads (BAM files) from each sample were used to calculate gene expression abundance at the whole-genome level using the featureCounts function provided by the Rsubread 2.18.0 R package.

### 3.4. Differential Expression Analysis

Raw counts were first filtered to remove lowly expressed genes using edgeR’s filterByExpr function (default parameters), and library sizes were normalized using the trimmed mean of M-values (TMM) method implemented in calcNormFactors. Gene-wise quasi-likelihood dispersions were estimated with estimateDisp, applying empirical Bayes moderation (shrinkage) toward a common trend, and differential expression was tested using the quasi-likelihood F-test (glmQLFit/glmQLFTest) implemented in the edgeR 4.2.1 R/Bioconductor package. Nominal *p*-values were adjusted for multiple testing using the Benjamini-Hochberg (BH) procedure, and genes with an adjusted *p*-value (FDR) < 0.05 and |log2FC| > 1 were considered significantly differentially expressed between polyps and normal adjacent tissue. Results were visualized using principal component analysis (PCA), hierarchical clustering heatmaps, and volcano plots. Genes exhibiting log2FC > 5 or <−5 (representing ≥32-fold changes) were identified as extreme differential expressors and examined independently.

### 3.5. Functional Enrichment Analysis

Functional annotation and pathway enrichment were performed using Gene Ontology (GO) and KEGG pathway analyses via the limma 3.58.x package, and gene-set enrichment analysis (GSEA) via clusterProfiler 4.12.6 R package/Bioconductor package. Biological process (BP). Normalized Enrichment Scores (NES) were calculated for each gene set; pathways with NES > 0 were considered activated and NES < 0 repressed (adjusted *p* < 0.05). Results were visualized as dot plots, bar charts, and Venn diagrams.

### 3.6. Cross-Dataset Comparison with TCGA Colorectal Cancer

To evaluate the overlap between JP epithelial differentially expressed genes (DEGs) and CRC-associated gene expression, we interrogated the TCGA colorectal adenocarcinoma dataset via UCSC Xena, encompassing 217 primary tumors and 32 adjacent normal samples. Genes upregulated or downregulated in both JP versus TCP and CRC versus normal tissue were identified by intersection. Survival analysis using disease-specific survival data was performed by Kaplan–Meier analysis using the median expression of selected genes as the stratification cutoff.

### 3.7. RT-qPCR Validation

Candidate genes were validated by reverse-transcription quantitative PCR (RT-qPCR) in six independent IEC samples from JP and paired TCP (distinct from those used for RNA-seq). Gene expression was normalized to *GAPDH*. Expression in CRC was assessed in seven paired tumor and peritumoral samples; IBD expression was evaluated in four ulcerative colitis (UC) and four Crohn’s disease (CD) samples (inflamed and non-inflamed zones), which were available as total mucosal biopsies rather than isolated IEC. Statistical comparisons were performed using the Wilcoxon signed-rank test (paired samples) with *p* < 0.05 considered significant.

## 4. Conclusions

The epithelial compartment from pediatric solitary juvenile colorectal polyps, associated with allergic sensitization, displays a comprehensive type 2 inflammatory transcriptome, including upregulation of the alarmin *IL33*, eosinophil-attracting chemokines, IgE receptor subunits, and leukotriene biosynthetic genes, alongside a profound shift in epithelial identity, barrier gene disruption, and metabolic reprogramming. The JP epithelial transcriptome partially overlaps with CRC-associated gene expression programs, with shared enrichment of epithelial and tissue remodeling pathways and suppression of oxidative metabolic processes. Rather than indicating premalignant transformation, this convergence may reflect conserved transcriptional programs associated with chronic epithelial stress, inflammation, and tissue remodeling. RT-qPCR validation in independent samples confirms that *CHI3L1* and *SERPINE1* are elevated in both JP and CRC, while *SERPINB4* appears JP-specific. Longitudinal, protein-level, and mechanistic studies are required to establish the clinical utility of these candidates and to determine whether the transcriptional features identified here carry prognostic significance for polyp recurrence or progression risk.

## Figures and Tables

**Table 1:** Differentially expressed genes associated with altered lineage identity, epithelial differentiation, stem cell programs, and barrier function in juvenile polyp epithelium.

Gene	log_2_FC	FDR	Biological Implication
**Up-Regulated Genes**			
**Altered Lineage Identity and Epithelial Differentiation**			
*MUC2*	1.32	0.021	Secretory mucin; upregulation reflects expansion of goblet/secretory cell lineage at the expense of absorptive enterocytes
*MUC5AC*	6.03	1.60 × 10^−5^	Gastric-type mucin not normally expressed in the colon; strong upregulation indicates metaplastic or stress-associated epithelial state
*LYZ*	3.37	5.70 × 10^−5^	Lysozyme; marker of Paneth-like secretory cells; upregulation consistent with secretory lineage expansion
*REG4*	4.59	1.70 × 10^−5^	Regenerating islet-derived protein 4; associated with Paneth-like and goblet cell secretory programs
*OLFM4*	2.66	0.002	Olfactomedin 4; marks immature or damage-associated regenerative epithelial progenitor state
**Stem Cell Niche and Signaling Disruption**			
*BMP4*	2.55	0.002	Bone morphogenetic protein 4; upregulated BMP ligand contributing to disruption of canonical BMP signalling and lineage commitment
*GREM1*	3.08	0.030	BMP antagonist; upregulation counteracts BMP signalling and disrupts normal crypt-villus axis patterning
**Barrier-Associated Gene Expression**			
*CLDN1*	2.22	0.006	Claudin-1; tight junction protein; upregulation may reflect compensatory or remodelling responses in the polyp epithelium
*CLDN2*	5.70	3.04 × 10^−4^	Claudin-2; pore-forming claudin that increases paracellular permeability; strong upregulation indicates impaired barrier integrity
**Down-Regulated Genes**			
**Altered Lineage Identity and Epithelial Differentiation**			
*CDX1*	−1.10	0.001	Caudal-type homeobox 1; transcription factor governing intestinal epithelial differentiation and enterocyte identity
*CDX2*	−1.16	0.002	Central regulator of enterocyte identity; maintains differentiated epithelial phenotype and activates absorptive marker genes (e.g., SI)
*VIL1*	−1.33	0.002	Villin-1; canonical absorptive enterocyte cytoskeletal marker; loss reflects reduced epithelial maturation
*ALPI*	−2.69	2.85 × 10^−4^	Alkaline phosphatase; absorptive enterocyte brush-border enzyme; downregulation consistent with loss of mature enterocyte identity
*CHGA*	−3.88	2.10 × 10^−5^	Chromogranin A; enteroendocrine cell marker; reduction indicates loss of enteroendocrine lineage representation
*DCLK1*	−1.76	0.009	Double cortin-like kinase 1; tuft cell marker; downregulation reflects reduced tuft cell lineage in polyp epithelium
**Intestinal Stem Cell Markers**			
*LGR5*	−2.10	0.005	Leucine-rich repeat-containing G-protein-coupled receptor 5; canonical intestinal stem cell marker in active crypts
*ASCL2*	−1.96	0.003	Achaete-scute homolog 2; transcription factor required for intestinal stem cell identity and Lgr5+ crypt maintenance
*SMOC2*	−3.50	2.69 × 10^−4^	SPARC-related modular calcium-binding protein 2; active stem cell niche marker co-expressed with LGR5
*PTK7*	−1.13	0.041	Protein tyrosine kinase 7; marks active and reserve stem cell populations in intestinal crypts
*LRIG1*	−1.16	5.83 × 10^−4^	Leucine-rich repeats and immunoglobulin-like domains 1; quiescent/reserve stem cell marker
*MEX3A*	−1.75	1.31 × 10^−4^	Mex-3 RNA binding family member A; marks slow-cycling, quiescent intestinal stem cells
*TERT*	−3.27	5.00 × 10^−6^	Telomerase reverse transcriptase; quiescent stem cell marker associated with long-term tissue self-renewal
**Notch and BMP Signaling Components**			
*HES1*	−1.52	2.16 × 10^−4^	Hairy and enhancer of split-1; canonical Notch transcriptional target regulating secretory vs. absorptive lineage specification
*BMP2*	−1.55	5.05 × 10^−4^	Bone morphogenetic protein 2; BMP ligand involved in enterocyte differentiation and crypt-villus axis maintenance
*ID2*	−1.04	9.00 × 10^−4^	Inhibitor of DNA binding 2; BMP-responsive transcription factor modulating intestinal lineage commitment
**Barrier-Associated Gene Expression**			
*CLDN3*	−1.14	0.030	Claudin-3; barrier-sealing tight junction protein; downregulation contributes to impaired epithelial barrier integrity
*CLDN4*	−1.26	0.005	Claudin-4; barrier-sealing tight junction protein; loss promotes paracellular permeability and antigen translocation
*CLDN23*	−2.57	9.90 × 10^−4^	Claudin-23; barrier-sealing claudin; downregulation, combined with CLDN2 upregulation, is consistent with disrupted barrier function

**Table 2:** Key differentially expressed genes associated with type 2 inflammation and allergic sensitization in JP versus TCP epithelium.

Gene	log_2_FC	FDR	Biological Implication
*SERPINB3*	12.16	8.34 × 10^−5^	Serine protease inhibitor implicated in epithelial protection, chronic inflammation, and allergic disease
*NMUR2*	10.08	6.87 × 10^−4^	Neuromedin U receptor 2; potential role in neuro-immune crosstalk at epithelial surfaces
*CHI3L1*	8.87	5.3 × 10^−5^	Chitinase-like lectin; marker of tissue remodeling, chronic inflammation, and allergic responses
*SERPINB4*	8.16	1.22 × 10^−4^	Serine protease inhibitor overexpressed in allergic conditions; modulates inflammation
*CCL3*	6.38	3.22 × 10^−4^	Pro-inflammatory chemokine (MIP-1α); recruits monocytes, lymphocytes, and neutrophils; linked to atopic disease
*PTGS2*	5.74	5.14 × 10^−5^	Cyclooxygenase-2; inducible enzyme regulating pro-inflammatory prostaglandin synthesis
*FCER1G*	5.59	1.35 × 10^−4^	Gamma subunit of the high-affinity IgE receptor; mediates allergic effector cell activation
*SERPINB2*	6.30	0.009	Plasminogen activator inhibitor 2; associated with type 2 inflammation and tissue injury
*TLR4*	5.14	4.84 × 10^−5^	Toll-like receptor 4; innate immune pattern-recognition receptor linked to allergen sensitization
*ALOX5AP*	4.51	3.65 × 10^−4^	5-lipoxygenase-activating protein; essential for leukotriene biosynthesis
*ALOX5*	3.85	2.94 × 10^−5^	5-lipoxygenase; key enzyme in arachidonic acid-derived leukotriene synthesis
*DPEP2*	4.89	2 × 10^−4^	Dipeptidase 2; metabolizes leukotrienes and modulates local inflammatory tone
*FCER1A*	2.42	0.046	Alpha subunit of the high-affinity IgE receptor
*CCL11*	3.52	0.005	Eotaxin-1; potent eosinophil chemoattractant; hallmark of type 2 inflammation
*CYSLTR2*	3.06	0.002	Cysteinyl leukotriene receptor 2; involved in allergic and inflammatory signaling
*IL33*	2.47	3.96 × 10^−4^	Epithelial-derived alarmin; initiates type 2 immunity via ILC2 and mast cell activation
*CCL22*	2.51	0.001	Th2 and Treg chemoattractant; promotes type 2 polarization
*CCL24*	2.10	3.04 × 10^−4^	Eotaxin-2; recruits eosinophils; associated with type 2 inflammation

**Table 3:** Differentially expressed genes in JP versus TCP that overlap with CRC-associated gene signatures.

Gene	log_2_FC (JP)	FDR	Biological Implication in CRC Context
*MMP3*	10.02	9.43 × 10^−5^	Matrix metalloproteinase; degrades ECM and activates other MMPs; facilitates invasion
*MMP1*	10.19	6.86 × 10^−5^	Collagenase; facilitates tumor invasion and metastasis
*TNFAIP6*	8.99	2.06 × 10^−4^	TNF-stimulated gene 6; ECM modulator and inflammation regulator; poor prognosis marker in CRC
*CXCL8*	8.10	9.54 × 10^−5^	IL-8; promotes tumor angiogenesis, immune exclusion, and metastatic dissemination
*FOXQ1*	5.96	4.44 × 10^−5^	Transcription factor linked to epithelial-to-mesenchymal transition and CRC metastasis
*ITGA5*	5.36	1.20 × 10^−5^	Integrin α5 subunit; regulates cell-ECM adhesion and migration; co-expressed with SERPINE1 in CRC
*TGFBI*	4.89	7.73 × 10^−5^	TGF-β-induced protein; modulates cell adhesion, ECM remodeling, and tumor microenvironment
*ICAM1*	4.67	1.20 × 10^−4^	Intercellular adhesion molecule; implicated in inflammation, leukocyte migration, and metastasis; poor prognosis marker
*SERPINE1*	4.42	4.92 × 10^−5^	PAI-1; promotes angiogenesis and tumor progression via P38-MAPK; poor prognosis in multiple cancers
*MMP19*	4.72	0.001	Matrix metalloproteinase; degrades ECM; linked to tumor invasiveness
*TIMP1*	3.75	1.26 × 10^−5^	Tissue inhibitor of metalloproteinases; paradoxically associated with poor prognosis in CRC
*ADAMTS4*	3.25	0.003	Aggrecanase; ECM remodeling; co-regulated with SERPINE1 in CRC
*IGF2BP3*	2.87	1.64 × 10^−5^	mRNA-binding protein promoting cell proliferation and survival; overexpressed in CRC
*TACSTD2*	2.35	0.001	Cell surface glycoprotein; marker of epithelial proliferation
*EMP3*	2.78	0.001	Membrane protein associated with cell proliferation and migration
*MMP14*	1.09	0.004	Membrane-type MMP; activates MMP2; poor prognosis marker in CRC

**Table 4:** Demographic and IgE sensitization profile of patients included in the study cohort.

Characteristic	Value
Patients, *n*	10
Age, years	2–6
Sex, male/female	6/4
History of rectal bleeding	10/10 (100%)
Juvenile polyp (JP) epithelial samples	8
Adjacent colonic tissue (TCP) epithelial samples	7
Patients contributing paired JP/TCP samples	7
Number of pedunculated and unique polyps	Sigmoid colon (5), rectum (2) and ascending colon (1)
Polyp size (cm)	0.5–3
Patients with total serum IgE data available	10/10
Total serum IgE, IU/mL	0.69–172.73 UI/mL
Patients with food-specific IgE data available	9/10
Food allergens evaluated	Cow’s milk, peanut, soy
IgE sensitization to ≥1 tested food allergen	9/9
IgE in polyps by flow cytometry	10/10 (100%)
Allergic symptoms	0/10 (0%)
Positive skin test (SPT)	0/10 (0%)

## Data Availability

The datasets generated and analyzed during the current study are available in the NCBI Sequence Read Archive (SRA) under BioProject accession number PRJNA1485045.

## Supplementary Materials

The following supporting information can be downloaded at: https://www.mdpi.com/article/10.3390/ijms27177946/s1.
